# Physiological activation of mGlu5 receptors supports the ion channel function of NMDA receptors in hippocampal LTD induction *in vivo*

**DOI:** 10.1038/s41598-018-22768-x

**Published:** 2018-03-13

**Authors:** Kenneth J. O’Riordan, Neng-Wei Hu, Michael J. Rowan

**Affiliations:** 1 Department of Pharmacology and Therapeutics and Institute of Neuroscience, Watts Building, Trinity College, Dublin, 2 Ireland; 2 Department of Gerontology, Yijishan Hospital, Wannan Medical College, Wuhu, China; 3 Department of Physiology and Neurobiology, Zhengzhou University School of Medicine, Zhengzhou, 450001 China

## Abstract

Synaptic long-term depression (LTD) is believed to underlie critical mnemonic processes in the adult hippocampus. The roles of the metabotropic and ionotropic actions of glutamate in the induction of synaptic LTD by electrical low-frequency stimulation (LFS) in the living adult animal is poorly understood. Here we examined the requirement for metabotropic glutamate (mGlu) and NMDA receptors in LTD induction in anaesthetized adult rats. LTD induction was primarily dependent on NMDA receptors and required the involvement of both the ion channel function and GluN2B subunit of the receptor. Endogenous mGlu5 receptor activation necessitated the local application of relatively high doses of either competitive or non-competitive NMDA receptor antagonists to block LTD induction. Moreover, boosting endogenous glutamate activation of mGlu5 receptors with a positive allosteric modulator lowered the threshold for NMDA receptor-dependent LTD induction by weak LFS. The present data provide support in the living animal that NMDA receptor-dependent LTD is boosted by endogenously released glutamate activation of mGlu5 receptors. Given the predominant perisynaptic location of mGlu5 receptors, the present findings emphasize the need to further evaluate the contribution and mechanisms of these receptors in NMDA receptor-dependent synaptic plasticity in the adult hippocampus *in vivo*.

## Introduction

Elucidation of the mechanisms of long-term potentiation (LTP) and long-term depression (LTD) provide a means of understanding synaptic plasticity processes that are believed to underlie information storage processes in learning and memory^1–3^. In the case of CA3-to-CA1 synapses in the hippocampus, LTD induction has been divided into NMDA receptor (NMDAR) and metabotropic glutamate receptor (mGluR) –dependent forms. Until recently, the ability of the ion channel of the NMDAR to conduct Ca^2+^ currents over prolonged periods was considered fundamental for the induction of NMDAR-dependent LTD by the standard low-frequency conditioning stimulation protocol (LFS, 1 Hz). Controversially, this view has been challenged recently by evidence that non-canonical, metabotropic, roles of the NMDAR mediate this form of LTD induction^4–6^. A major other form of synaptically evoked hippocampal LTD, usually induced by paired-pulse LFS, is mGluR-dependent and has been reported to operate via distinct signaling pathways^7–9^. However, this apparent dichotomy may only be valid under conditions where either one or other of these glutamate receptors is activated alone^9,10^. Indeed, it has been known for some time that the magnitude of exogenous mGlu5R agonist-induced LTD is dependent on the level of background NMDAR tone^11^. Since mGlu5Rs are predominantly located outside the synapse, especially perisynaptically within ~60 nm of the synapse edge^12–15^ and form part of an extensive signaling interactome with NMDARs^16^, if sufficient glutamate concentration accumulates at perisynaptic sites during LTD induction, significant cross-talk between these two receptors and their downstream signaling mechanisms is likely.

Presumably because of the known difficulty of inducing LTD in the intact hippocampus *in vivo* of adult animals^17,18^ most research on this topic has been performed in brain slices from young animals. Recently, we reported that high-intensity electrical LFS (LFS) reliably induced robust LTD in the hippocampus of anaesthetized rats^19^. In contrast to most previous *in vitro* studies (e.g. see^10^), the induction of this *in vivo* LTD was resistant to block by standard doses of either NMDAR or mGlu5R antagonists. Because LFS-evoked synaptically released glutamate will spillover to activate peri- and extra-synaptic glutamate receptors^20,21^, and therefore is likely to co-activate both mGlu5R and NMDARs, we wondered if an interaction between these receptors shaped the induction of LTD.

Therefore, we decided to revisit the glutamate receptor requirements for the induction of synaptic LTD *in vivo*. We found that relatively high doses of locally-injected NMDAR antagonists were required to block LFS-induced LTD. We show that mGlu5R activation by endogenously released glutamate facilitated the NMDAR-dependent LTD and made it resistant to block with standard doses of NMDAR antagonists. Moreover, the GluN2B subunit and a functional ion channel in NMDARs were obligatory for such LTD induction *in vivo*.

## Results

### Role of NMDA and mGlu5 receptors in the induction of hippocampal synaptic LTD by low-frequency conditioning stimulation *in vivo*

**Figure 1:**
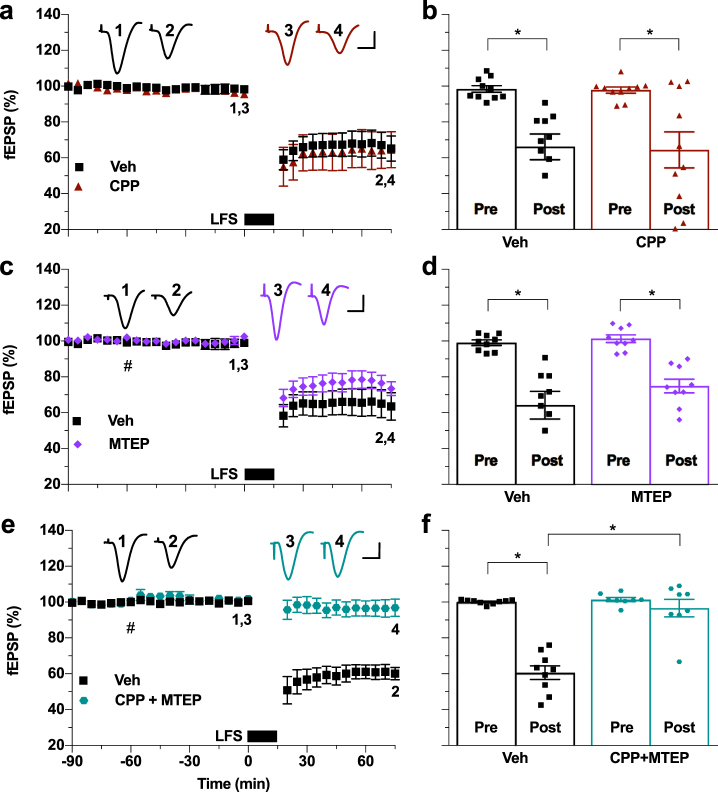
Combined administration of antagonists of NMDA and mGlu5 receptors, when given systemically, is necessary to prevent the induction of hippocampal synaptic LTD by low-frequency conditioning stimulation *in vivo*. (**a**) The competitive NMDAR antagonist CPP (10 mg/kg, i.p.), injected alone 2.25 h prior to electrical LFS (900 high-intensity pulses at 1 Hz) (see also Supplementary Fig. S1), had no significant effect on LTD induction (CPP: 64.4 ± 10%, mean ± SEM fEPSP amplitude expressed as a % of the pre-LFS baseline, n = 10; controls: 66.2 ± 7.1%, n = 10; *P* < 0.05 both compared with respective pre-LFS baselines, *P* > 0.05 between groups; two-way ANOVA RM-Sidak). (**b**) Summary of the mean EPSP amplitude data in (a) before (pre) and 1 h after (post) application of LFS. (**c**) Injection of the mGlu5R negative allosteric modulator MTEP (3 or 6 mg/kg, i.p., hash) alone did not significantly alter the induction of LTD by LFS 1 h later (MTEP: 75.0 ± 3.8%, n = 9; controls: 64.3 ± 7.7%, n = 9; *P* < 0.05 both compared with baselines, *P* > 0.05 between groups, two-way ANOVA RM-Sidak). (**d**) Summary of the mean EPSP amplitude data in (c). (**e**) Combined administration of CPP (10 mg/kg, i.p., 2.25 h pre-LFS) with MTEP (3 mg/kg, i.p., 1 h pre-LFS, hash), blocked induction of LTD (CPP + MTEP: 96.7 ± 4.9%, *n* = 8, *P > *0.05 compared with pre-LFS baseline; Veh: 60.6 ± 3.8%, *n* = 9, *P* < 0.05 compared with baseline and between groups; two-way ANOVA RM-Sidak). (**f**) Summary of the mean EPSP amplitude data in (**e**). *P < 0.05. Insets show typical fEPSP traces at the times indicated. Calibration bars: vertical, 1 mV; horizontal, 10 ms.

Because we had previously found that the induction of LTD of electrically evoked field excitatory postsynaptic potentials (fEPSPs) by LFS, consisting of 900 high-intensity pulses at 1 Hz, was not blocked by standard systemic doses of either NMDAR or mGlu5R antagonists when applied alone^19^, we wondered if both types of receptors needed to be blocked simultaneously. Consistent with our previous finding^19^, using a dose (10 mg/kg, i.p.) of the competitive NMDAR antagonist CPP known to completely inhibit synaptic LTP in the CA1 area^22^, the application of LFS after injection of CPP induced robust and stable synaptic LTD similar in magnitude to that induced in control, vehicle-injected rats (Fig. 1a,b). Again, confirming our previous report^19^, application of LFS in MTEP-treated rats induced robust and stable LTD. Initially we tested the standard dose of MTEP (3 mg/kg, i.p., *n* = 5) which is known to non-competitively block mGlu5Rs, with a rat hippocampal receptor occupancy of >90%^23^. When this dose proved ineffective we doubled the dose (*n* = 4) with similar results, so we pooled the data for the two doses (Fig. 1c,d). Intriguingly, even though neither of these treatments on their own affected LTD, combined systemic injection of these antagonists completely abrogated electrical induction of synaptic LTD (Fig. 1e,f).

**Figure 2:**
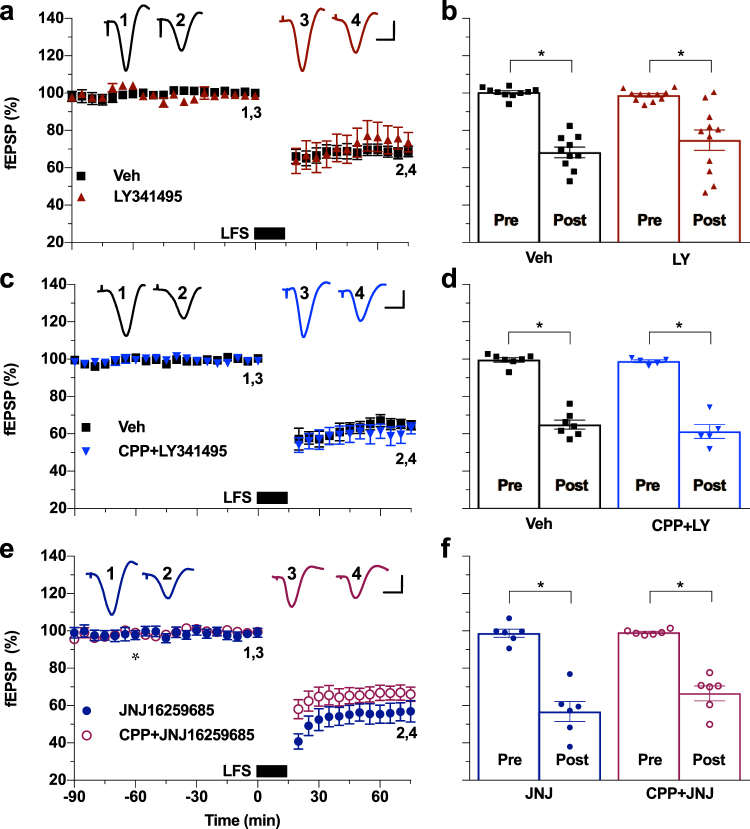
Group II mGluR or mGlu1R antagonists alone or in combination with NMDAR blockade fails to inhibit synaptic LTD *in vivo*. (**a**) The potent group II mGluR antagonist LY341495 (3 mg/kg, i.p.) did not significantly affect the induction of LTD (LY341495: 74.8 ± 5.4%, *n* = 11; Veh: 68.2 ± 2.9%, *n* = 10; *P* < 0.05 both compared with baselines, *P > *0.05 between groups; two-way ANOVA RM-Sidak) (see also Supplementary Fig. S1). (**b**) Summary of the mean EPSP amplitude data in (a). (**c**) Intraperitoneal administration of CPP (10 mg/kg, 2.25 h pre-LFS) combined with LY341495 (3 mg/kg, 1 h pre LFS), had no effect on LTD (CPP + LY341495: 61.2 ± 3.7%, n = 5; Veh: 64.9 ± 2.4%, n = 7, *P* < 0.05 compared to respective baselines and *P* > 0.05 between groups; two-way ANOVA RM-Sidak). (**d**) Summary of the mean EPSP amplitude data in (c). (**e**) Neither injection of the selective mGlu1R antagonist JNJ16259685 (0.5 mg/kg, s.c., asterisk) alone, nor when combined with CPP (10 mg/kg, i.p., 2.25 h pre-LFS) significantly affected the induction of LTD (JNJ16259685: 63.1 ± 1.8%, n = 6; CPP + JNJ16259685: 66.6 ± 4.0%, n = 6; *P* < 0.05 both compared with baselines, *P > *0.05 between groups; two-way ANOVA RM-Sidak). (**f**) Summary of the mean EPSP amplitude data in (e). *P < 0.05. Calibration bars: vertical, 1 mV; horizontal, 10 ms.

These findings indicate that LFS increases glutamate concentration sufficiently to activate mGlu5Rs, which are found predominantly outside, especially on the edge of, the synapse^12–15^. Co-activation of mGlu5Rs with NMDARs located in this vicinity is known to strongly enhance the function of the NMDARs^24–26^. Group II mGluRs also are located perisynaptically^27^, can enhance NMDA-evoked currents^28,29^, and have been implicated in the induction/maintenance of LTD^30–32^. However, unlike mGlu5Rs, they do not appear to have a role during LTD induction by LFS *in vivo*. Thus, the high potency mGlu2/3 R antagonist LY341495 (3 mg/kg, i.p.) failed to significantly affect the magnitude of LTD when administered alone (Fig. 2a,b), or when co-administered with CPP (Fig. 2c,d).

Like mGlu5Rs, mGlu1Rs have been implicated in LTD and are located peri- and extra-synaptically^7,33,34^. The selective mGlu1R antagonist JNJ16259685 (0.5 mg/kg, s.c.), when injected alone or in combination with CPP (10 mg/kg, i.p.), failed to significantly affect the magnitude of LTD (Fig. 2e,f).

**Figure 3:**
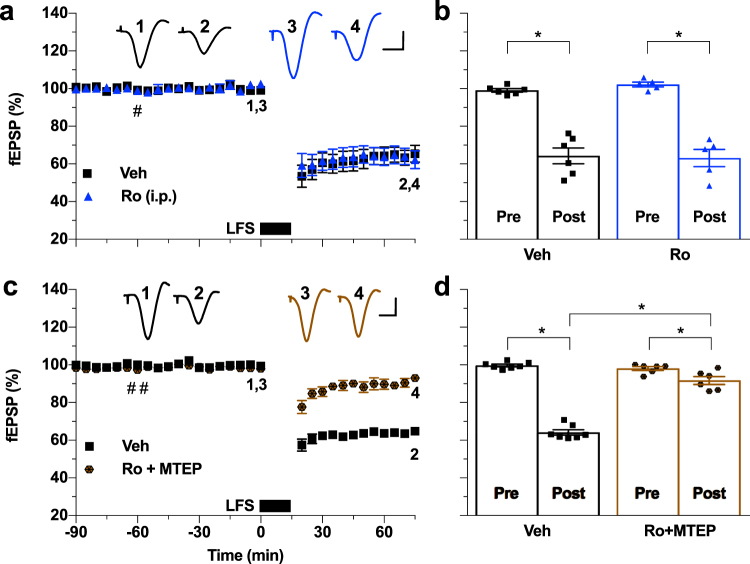
GluN2B-subunit-selective antagonist, systemically administered with an mGlu5R antagonist, prevents LFS induction of LTD. (**a**) The non-competitive GluN2B subtype selective NMDAR antagonist Ro 25-6981 (12 mg/kg, i.p., 1 h pre-LFS, hash) alone had no effect on LTD induction (Ro 25-6981: 63.1 ± 4.6%, *n* = 5; Veh: 64.3 ± 4.2%, *n* = 6; *P* < 0.05 both compared with baselines, *P* > 0.05 between groups; two-way ANOVA RM-Sidak). (**b**) Summary of the mean EPSP amplitude data in (a). (**c**) In contrast, a combination of Ro 25-6981 (12 mg/kg, i.p., first hash) with MTEP (3 mg/kg, second hash) strongly attenuated LTD (Ro 25-6981 + MTEP: 91.7 ± 2.1%, *n* = 6; Veh: 64.2 ± 1.4%, *n* = 7; *P* < 0.05 compared with respective baselines and between groups; two-way ANOVA RM-Sidak). (**d**) Summary of the mean EPSP amplitude data in (**c**). *P < 0.05. Calibration bars: vertical, 1 mV; horizontal, 10 ms.

Whereas group II mGluRs enhance GluN2A subunit containing NMDAR function^28^, mGlu5R enhancement is likely achieved at least partly by promoting the phosphorylation of GluN2B subunits with consequent increased membrane stabilization of NMDARs^35^. Therefore, we tested the ability of a non-competitive GluN2B-selective antagonist, the negative allosteric modulator Ro 25-6981, to prevent LTD induction in the presence and absence of MTEP. Consistent with the CPP results, whereas Ro 25-6981 (12 mg/kg, i.p) alone failed to significantly alter LTD (Fig. 3a,b) the same dose of Ro 25-6981 given together with MTEP, greatly reduced the magnitude of LTD (Fig. 3c,d).

These findings are consistent with the supposition that the interaction between mGlu5R and GluN2B subunit-containing NMDARs facilitates the induction of NMDAR-dependent LTD. However, an alternative potential explanation of these data is that the activation of either mGlu5Rs or NMDARs by LFS can independently mediate the induction of LTD *in vivo*.

### Absolute requirement for GluN2B-subunit-containing NMDARs during LTD induction by LFS *in vivo*

**Figure 4:**
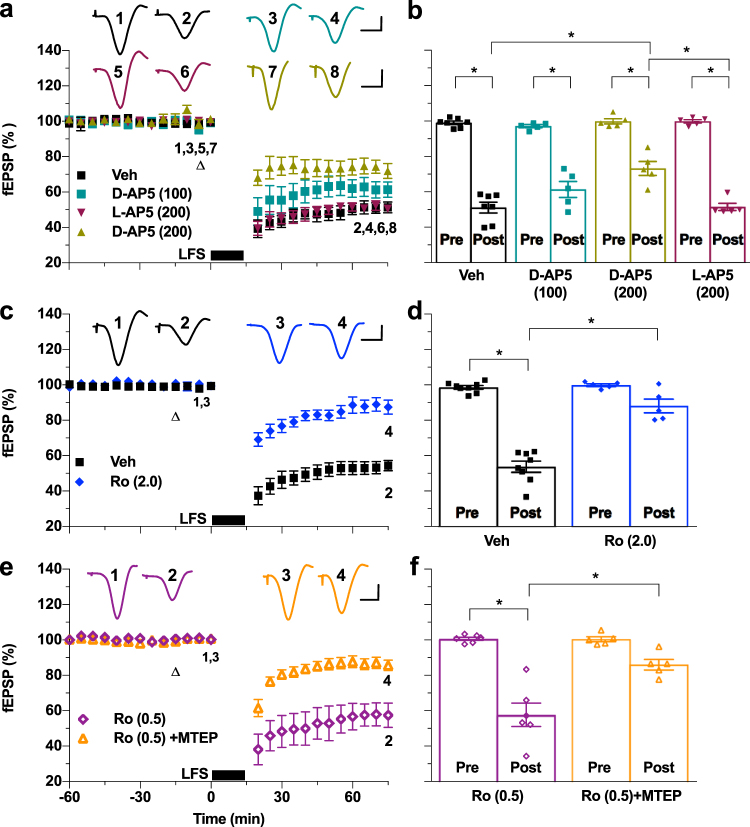
Relatively high doses of locally-injected NMDAR antagonists prevent LTD induction by LFS *in vivo*. (**a**) Intracerebroventricular administration of 200 nmol of the competitive NMDAR antagonist D-AP5 (triangle), 10 min before LFS-900 attenuated LFS induced LTD (200 nmol D-AP5: 73.2 ± 3.9%, n = 5; *P* < 0.05 compared with Veh: 51.1 ± 3.0%, n = 7; and *P* < 0.05 compared with 200 nmol of the NMDAR low-affinity stereoisomer of D-AP5, L-AP5: 51.4 ± 2.1%, n = 5; one-way ANOVA-Sidak). The magnitude of LTD in animals injected with the lower dose 100 nmol D-AP5 was not significantly different from vehicle or 200 nmol D-AP5 (100 nmol D-AP5; 61.3 ± 4.5%, *n* = 5). All groups were significantly different from their respective baselines (paired *t*). (**b**) Summary of the mean EPSP amplitude data in (a). (**c**) Furthermore, i.c.v. application of the non-competitive Ro 25-6981 (2 nmol, triangle) strongly inhibited LTD (2 nmol Ro: 88.2 ± 3.9%, *n* = 5, *P* > 0.05 compared with baseline; Veh: 53.8 ± 3.1%, *n* = 8, *P* < 0.05 compared with baseline and between groups; two-way ANOVA RM-Sidak). (**d**) Summary of the mean EPSP data in (c). (**e**) I.c.v. injection of the lower dose Ro 25-6981 (0.5 nmol, triangle) alone did not affect LTD induction but completely prevented LTD when given in combination with MTEP (3 mg/kg, i.p., 1 h pre-LFS) (0.5 nmol Ro: 57.7 ± 6.6%, *n* = 6, *P* < 0.05 compared with baseline; 0.5 nmol Ro + MTEP: 86.4 ± 3.0%, *n* = 5, *P* > 0.05 compared with baseline, *P* < 0.05 between groups; two-way ANOVA RM-Sidak). (**f**) Summary of the mean EPSP amplitude data in (e). *P < 0.05. Calibration bars: vertical, 1 mV; horizontal, 10 ms.

We hypothesized that, if endogenously released glutamate during LFS activates mGlu5Rs to increase the number of functional NMDARs available for LTD induction, then the dose of NMDAR antagonist required to block LTD should be greater when administered alone, relative to when co-administered with an mGlu5R antagonist. In order to assess this possibility, rather than inject higher doses systemically which lead to increased risk of respiratory depression, we injected relatively high doses of NMDAR antagonists locally near the hippocampus, via the intracerebroventricular (i.c.v.) route, thereby minimizing the likelihood of significant systemic toxicity. We started by testing the effect of i.c.v. injection of the competitive antagonist D-AP5. Whereas a dose (100 nmol) that inhibits LTP induction^36^ was without significant effect, double this dose partially attenuated the magnitude of LTD (Fig. 4a,b). As an additional control, we tested the same dose (200 nmol) of L-AP5, the AP5 stereoisomer that is relatively inactive at NMDARs^37^, and found that it failed to affect LTD, consistent with a crucial role for NMDARs in LFS induction of LTD.

Given that, compared with LTP, NMDAR-dependent ‘paired-burst’ –induced LTD was reported to be preferentially inhibited by antagonists of GluN2B over GluN2A *in vivo*^38,39^ and the known importance of GluN2B subunit-containing NMDARs in mediating facilitatory effects of mGlu5R co-activation^35^, next we tested the effect of local injection of the very selective GluN2B antagonist Ro 25-6981 on its own. Indeed, treatment with a relatively high dose (2 nmol, i.c.v.) of Ro 25-6981 greatly diminished the magnitude of LTD induced by LFS, compared with vehicle-treated animals (Fig. 4c,d). Furthermore, Ro 25-6981, at the lower dose of 0.5 nmol that was without significant effect on LTD when given on its own, inhibited LTD when given in combination with MTEP (Fig. 4e,f).

GluN2A subunit-containing NMDARs may also be required for LTD^40,41^ and mGlu5Rs couple to Gαq subunit-containing G proteins that can enhance GluN2A NMDAR function^41^. Moreover, activation of mGlu5Rs has been reported to promote the tyrosine phosphorylation of both GluN2A and GluN2B subunits in hippocampal cultures^42^ and recombinant GluN2A NMDAR-mediated Ca^2+^ responses can be enhanced by stimulation of mGlu5R, in a tyrosine kinase-dependent manner^43^. Therefore we tested the effect of local i.c.v. injection of the GluN2A-preferring antagonist NVP-AAM077 (NVP). Treatment with NVP (0.5 or 1 nmol) completely blocked HFS-induced LTP (Supplementary Fig. S2a,b). However, NVP failed to affect the magnitude of LTD induced by LFS, when injected alone (1 nmol) or when combined (0.5 or 1 nmol) with the mGlu5R antagonist MTEP (Supplementary Fig. S2c,d).

We didn’t test the effect of high local dose of MTEP on LTD induction because systemic administration of the doses employed in the previous section (see above) achieve very high receptor occupancy^23^.

### LFS-evoked endogenous glutamate release activates mGlu5Rs to facilitate the induction of LTD

**Figure 5:**
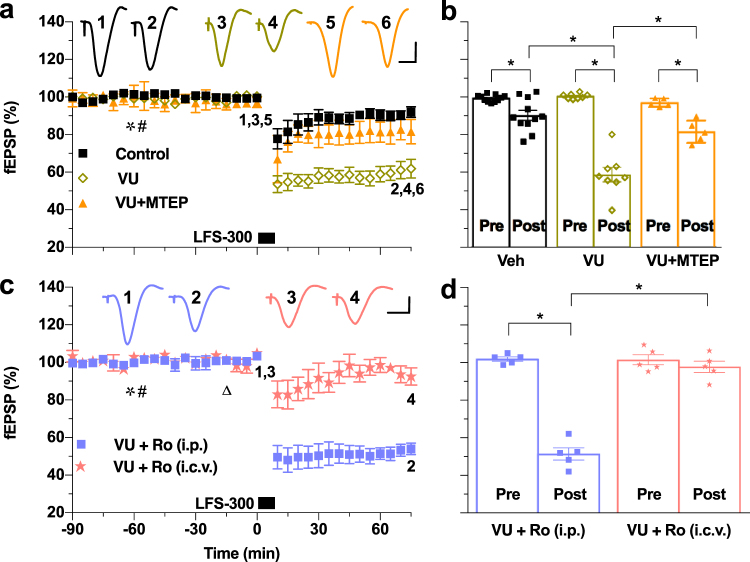
Endogenous glutamate release activates mGlu5Rs to facilitate the induction of LTD *in vivo*. (**a**) The mGlu5R positive allosteric modulator VU 0360172 (15 mg/kg, s.c., asterisk) facilitated the induction of LTD by a peri-threshold electrical LFS-300 conditioning protocol (300 high-intensity pulses at 1 Hz) relative to vehicle controls (control: 90.1 ± 2.6%, n = 11; VU 0360172: 58.7 ± 4.0%, n = 8, *P* < 0.05 compared with baselines and between groups; one-way ANOVA-Sidak followed by paired *t*). The facilitation of LTD by VU 0360172 was prevented when the animals were co-treated with MTEP (3 mg/kg, i.p., 1 h pre-LFS, hash) (VU + MTEP: 81.4 ± 2.7%, n = 5; *P* < 0.05 compared with VU group). (**b**) Summary of the mean EPSP amplitude data in (a). (**c**) Local injection of a relatively high dose (2 nmol, i.c.v., triangle) of the GluN2B antagonist Ro 25-6981 prevented LTD induction by LFS-300 in the presence of VU 0360172 (asterisk), whereas systemic treatment (12 mg/kg, i.p., hash) did not (i.c.v. Ro 25-6981: 97.8 ± 3.0%, n = 5, *P* > 0.05 compared with baseline; i.p. Ro 25-6981: 51.4 ± 3.3%, n = 5, *P* < 0.05 compared with baseline and between groups; two-way ANOVA RM-Sidak). (**d**) Summary of the mean EPSP amplitude data in (c). *P < 0.05. Calibration bars: vertical, 1 mV; horizontal, 10 ms.

The findings described above support the hypothesis that mGlu5R and GluN2B NMDAR co-activation during LFS enhances LTD induction. Therefore, we wondered if pharmacologically boosting endogenous glutamate activation of mGlu5Rs would facilitate the induction of LTD. We predicted that a positive allosteric modulator of these receptors should lower the threshold for LTD, enabling its induction by weak LFS. Therefore, we examined the effect of the mGlu5R positive allosteric modulator VU 0360172 (15 mg/kg, s.c.) on the ability of a peri-threshold induction protocol (300 high-intensity pulses at 1 Hz, LFS-300) to induce LTD. Consistent with the hypothesis, VU 0360172 facilitated the induction of robust LTD by the peri-threshold electrical LFS-300 in a manner that was inhibited by MTEP (Fig. 5a,b).

The data presented so far indicate that the relatively low potency of competitive and non-competitive NMDAR antagonists, when given on their own, in blocking LTD induction by LFS might be caused by co-activation of mGlu5Rs. To further test this hypothesis we compared the ability of systemic and local injection with the non-competitive GluN2B antagonist Ro 25-6981 to prevent LTD induced by weak LFS in the presence of the mGlu5R positive allosteric modulator. Whereas the induction of LTD by LFS-300 in the presence of VU 0360172 was not blocked by a standard systemic dose (12 mg/kg, i.p.) of Ro 25-6981, local high-dose (2 nmol, i.c.v.) injection of this antagonist completely blocked this form of LTD (Fig. 5c,d).

The observed positive allosteric mGlu5R modulator-mediated facilitation of LTD induction, and the requirement for high-dose local GluN2B antagonist to block this facilitated LTD provide complimentary support for the involvement of mGlu5-NMDA receptor co-activation in LFS-induced LTD.

### NMDAR ion channel function is required for the induction of LTD *in vivo*

**Figure 6:**
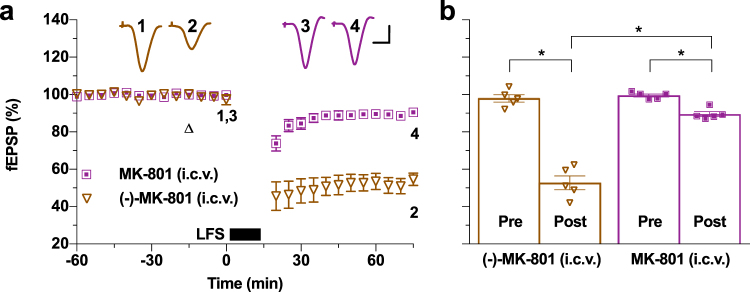
Necessity for NMDAR ion channel function in the induction of LTD *in vivo*. (**a**) A relatively high dose of locally-injected use-dependent NMDAR ion channel blocker MK-801 (60 nmol, i.c.v., triangle), in comparison with the control (−) stereoisomer, strongly inhibited LTD induction *in vivo* (i.c.v. MK-801: 89.5 ± 1.3%, *n* = 5; i.c.v.(−)-MK-801: 52.8 ± 3.7%, *n* = 5, *P* < 0.05 compared with baselines and between groups; two-way ANOVA RM-Sidak). (**b**) Summary of the mean EPSP amplitude data in (c). *P < 0.05. Calibration bars: vertical, 1 mV; horizontal, 10 ms.

In the light of the controversy over the requirement for ion flux through the NMDAR channel in LFS induction of synaptic LTD *in vitro*^4–6^, next we examined the effect of the use-dependent NMDAR channel blocker MK-801 *in vivo*. In order to reduce the likelihood of significant systemic toxicity, we tested the effects of local injection of MK-801 in the brain. We found that MK-801 (60 nmol, i.c.v.) prevented LTD induction by electrical stimulation in a stereoselective manner. Consistent with a need for the ion channel function of the NMDARs in the induction of LTD, only the NMDAR-selective (+)-enantiomer (MK-801) reduced the magnitude of LTD whereas the control (−)-enantiomer (60 nmol) appeared inactive (Fig. 6a,b).

These data provide strong evidence that the induction of synaptic LTD *in vivo* requires ion flux via NMDARs.

## Discussion

In the present study, we have re-examined glutamate receptor mechanisms underlying the induction of LTD by LFS at CA3-to-CA1 synapses in the hippocampus *in vivo*. We discovered that local application of relatively high doses of competitive and non-competitive receptor antagonists, when given alone, was required to reveal the NMDAR-dependence of synaptic LTD induction. Remarkably, although mGlu5R antagonism alone failed to affect LTD, standard systemic doses of NMDAR antagonists were sufficient to prevent LFS-induced LTD when mGlu5Rs were simultaneously blocked. Complementing these findings, a positive allosteric modulator of mGlu5Rs lowered the threshold for electrically-induced LTD. Moreover, local application of a relatively high dose of NMDAR antagonist was again required to inhibit this mGlu5R positive allosteric modulator-facilitated LTD. We also found strong evidence of the obligatory requirement for the GluN2B subunit and the ion channel function of NMDARs in the induction of LTD by LFS *in vivo*. Taken together these findings attest that endogenously released glutamate activation of mGlu5Rs enhances LTD induction that requires the GluN2B subunit and ion channel function of NMDARs.

The induction of LTD by LFS was inhibited only by local application of relatively high doses of a variety of NMDAR antagonists that act via different mechanisms: (1) bind competitively to the orthosteric glutamate binding site (D-AP5), (2) non-competitively block the GluN2B subunit (negative allosteric modulator Ro 25-6981) or (3) non-competitively block the open ion channel (MK-801). The lack of significant inhibitory effect of standard doses of these and similar agents previously, led us to wrongly assume that high-intensity LFS induction of synaptic LTD *in vivo* is NMDAR-independent^19^. These results reinforce the need to reassess LTD induced by other electrical stimulation protocols that are currently considered by many not to require NMDAR activation, on a case by case basis^9,44,45^, see also^46^. This requirement for local application of relatively high doses of NMDAR antagonists is unlikely to be solely because LFS increases glutamate release, since the standard doses of D-AP5 and CPP used here, that failed to inhibit LTD, completely block the induction of LTP by electrical high-frequency conditioning stimulation that greatly increases glutamate release^36,47^. Moreover, by definition, non-competitive blockade of NMDARs will be relatively independent of ambient glutamate concentration especially at synapses with low receptor reserve. The requirement for a relatively high concentration of antagonist to achieve significant block of LTD induction therefore could be caused by a recruitment of additional functional NMDAR numbers, perhaps as a consequence of glutamate spillover to extrasynaptic NMDARs which may be preferentially blocked by GluN2B selective antagonists^21,48^, but see^49^. Our finding that blocking mGlu5Rs lowered the dose of NMDAR antagonist required to inhibit LTD by LFS is consistent with, but does not prove, the interpretation that mGlu5R co-activation is critically involved. Previously NMDAR-dependent LTD induction in rats was reported to be blocked by i.c.v. injection of either D-AP5 or the mGlu5R antagonist MPEP alone^50^. The apparent differences from our findings may be caused by different recording (freely behaving versus anesthetized) or stimulation (high-intensity LFS used here) conditions. Moreover MPEP, unlike MTEP, can also block NMDARs if the local concentration reaches above ~10 µM^23,51,52^. Interestingly, mGlu5 and NMDA receptors are associated as part of an interactome^16^ and co-activation of these receptors enhances NMDAR-mediated synaptic function^24–26^. Moreover, a PKC-dependent activation by mGlu1Rs leading to increased numbers of functional NMDA receptors and increased mean channel open time has been proposed as a basis for modulating synaptic plasticity^53–55^. Although we did not find evidence for a role of mGlu1R, since activation of mGlu5R also increases PKC activation, similar modulatory mechanisms may apply for this receptor subtype. Previous research on hippocampal slices from young rats found that although LFS induced LTD of the NMDAR-mediated component of synaptic transmission required mGlu1R activation, LTD of the AMPAR-mediated component was not^34^ (see also refs^7,45,56,57^). Further, complementary, confirmation of the role of mGlu5Rs in the direct regulation of NMDAR function *in vivo* was our finding that a positive allosteric modulator at mGlu5Rs lowers the threshold for the induction of LTD by LFS. Importantly, similar to LTD induced by standard LFS, relatively high-dose NMDAR antagonist also was required to inhibit this pharmacologically potentiated LTD. Although it is possible that mGlu5R-mediated depolarization^58^ or dis-inhibition^59^ may be involved in the facilitation of LTD, it is unclear how such an action would increase the dose requirement for non-competitive NMDA receptor antagonist to block LTD. Future research, including high-resolution confocal microscopy *in vivo*, will be necessary to directly assess if mGlu5R activation boosts functional NMDAR numbers or possibly interacts at the level of downstream mediators such as P38 MAP kinase which has been implicated in mediating both mGluR- and NMDAR-dependent LTD^1,60^.

Recent research^35^ emphasizes a key role for GluN2B subunits in mediating mGlu5R enhancement of NMDAR excitability, where co-activation of mGlu5R/NMDAR activates Src kinases, synergistically leading to GluN2B(Tyr1472) phosphorylation. Similar phosphorylation prevents GluN2B internalization, stabilizing GluN2B-containing NMDARs in the plasma membrane^61,62^. In striatal neurons activation of mGlu5R dissociates CaMKIIα from the receptor thereby promoting its binding to any adjacent GluN2B subunit, which enables CaMKIIα to phosphorylate GluN2B^63^. Such phosphorylation stabilizes GluN2B in the membrane and enhances NMDAR function^63,64^. Indeed our data demonstrate that GluN2B subunits are critical for LTD induction by LFS, consistent with previous *in vivo*^38,65^, and many *in vitro* studies, e.g.^66,67^, reviewed in^40^ but see^68–70^. Similarly, although an apparent resistance of LTD to inhibition by NMDAR antagonists, including D-AP5 and CPP, *in vitro* has been attributed to a preferential involvement of GluN2C/D subunit-containing NMDARs in juvenile rat hippocampus^71,72^ the present data strongly indicate a requirement for GluN2B in LTD in the adult rat *in vivo*. GluN2 subunit expression and localization changes markedly across development, with a higher ratio of GluN2A:GluN2B, and a shift of GluN2B from synaptic to a predominantly perisynaptic and extrasynaptic location, in adulthood^73^. Small changes in age during development affect the magnitude or even the presence of LTD^74^, consistent with a critical role for GluN2B in the induction of LTD. Based on our findings with Ro 25-6981, GluN1/GluN2B diheteromers are required for synaptic LTD induced by high-intensity LFS in the adult rat *in vivo*. The finding that a dose of NVP that completely inhibited LTP, given alone did not affect LFS-induced LTD, is consistent with previous *in vivo* findings that LTP is more sensitive to NVP than paired burst-induced LTD^38,39^. However, it does not rule out roles for GluN1/GluN2A diheteromers or GluN1/GluN2A/GluN2B triheteromeric NMDARs^40,41,75–78^. The inability of a relatively low dose of NVP to inhibit LTD when given in combination with MTEP contrasts with our finding of a lower dose requirement for Ro 25-6981 in the presence of MTEP. Future research should determine if GluN2A and GluN2B differentially couple to mGlu5Rs in the adult rat hippocampus.

The ability of the open-channel, use-dependent, blocker of NMDARs MK-801^49^, to inhibit LTD strongly indicates that the LTD induction is dependent on ion flux through NMDARs. The widely accepted view that the faster and greater Ca^2+^ influx through NMDARs during high-frequency stimulation protocols leads to LTP at hippocampal synapses while slower and smaller Ca^2+^ influx through NMDARs results in LTD, has been challenged recently^6,79^. Significantly, although we did not examine the requirement for a metabotropic function of NMDARs, the present study showing that LTD was blocked by MK-801strongly supports the view that Ca^2+^ influx through NMDARs is required for LTD induction *in vivo*. Thus, although our data are consistent with a physical interaction between mGlu5Rs and GluN2B facilitating LTD induction, the obligatory involvement of NMDAR ion channel function in triggering LTD *in vivo* emerges as paramount.

In view of the predominant localization of mGlu5Rs at peri- and extra-synaptic sites^12–15^, our finding of a facilitatory role of mGlu5Rs in NMDAR-dependent LTD induction is consistent with the interpretation that significant co-activation of mGlu5Rs and NMDARs at non-synaptic sites is involved. The likelihood of this occurring will be dependent on the spatial pattern of synaptic activation, with electrical field stimulation, as used in the present experiments, providing highly favorable conditions for glutamate spillover-mediated co-activation of mGlu5 and NMDA receptors, whereas NMDAR-dependent LTD induced by relatively sparse optogenetic stimulation appears to be independent of mGlu5Rs^80^. Nearby synapses are especially likely to be co-activated under pathological conditions such as epilepsy and spillover to neighboring synapses will be increased when glutamate homeostasis is compromised, such as occurs in psychiatric and neurological illnesses. Undoubtedly, hippocampal mGlu5Rs act as sentinels of glutamate spillover and, when activated in concert with GluN2B, trigger a persistent down-regulation of potentially redundant or inappropriate synaptic transmission, thus placing mGlu5R facilitation of LTD in the centre of the synaptic physiology-pathology continuum.

## Materials and Methods

### Animal surgery, electrode and cannula implantation

Animal care and experimental protocols were carried out in accordance with the approval of the Health Products Regulatory Authority, Ireland. Adult male Wistar and Lister hooded rats, supplied by Trinity College Comparative Medicine, were housed in a 12-h light-dark cycle at room temperature (19–22 °C). Electrode implantation/recording was performed on 4.5–6 month-old (400–500 g) animals, anaesthetized with urethane (1.5–1.6 g/kg, i.p.) and core body temperature was maintained at 37 °C while placed in a stereotaxic apparatus for the duration of the experiment. A twisted bipolar Teflon-coated tungsten wire (inner core diameter 50 μm, external diameter 75 µm) stimulating electrode was lowered into the ipsilateral stratum radiatum of area CA3 at coordinates −4.2 mm posterior to the coronal suture, and −3.8 mm lateral to the sagittal suture, measured from bregma. In some animals, which had also previously undergone surgery to transduce channelrhodopsin in CA3 cells, a 200 μm optical fiber cable was also attached to the stimulating electrode, but the light responses were unsuitable for recording. A monopolar Teflon-coated tungsten wire (inner core diameter 75 μm, external diameter 112 μm) was lowered into the stratum radiatum of area CA1 at coordinates −3.5 mm posterior to the coronal suture, and −2.5 mm lateral to the sagittal suture, measured from bregma. Screw electrodes located over the contralateral cortex were used for reference and earth. The final placement of electrodes was optimized by using electrophysiological criteria and confirmed via post-mortem analysis.

Where necessary, a stainless-steel cannula (22 gauge, 0.7 mm outer diameter) was implanted above the ipsilateral-to-recording ventricle (0.5 mm posterior to the coronal suture, and 1 mm lateral to the sagittal suture, lowered 4.2 mm from the surface of the skull) and secured in place with dental cement. The solutions were injected through an internal cannula (28 gauge, 0.36 mm outer diameter) no quicker than 1 μl per min. Verification of the placement of the cannula was performed post-mortem.

### *In vivo* recording

**S**ingle square-wave electric pulse (0.2 ms duration) test stimulation was delivered to the Schaffer-collateral/commissural pathway every 30 s, at a frequency of 0.033 Hz, to evoke fEPSPs at a low intensity until stabilisation of a baseline response. Input/output (i/o) curves were generated for each rat separately to ascertain half maximum stimulation intensity that was used for test pulse stimulation. Baseline transmission had to be stable for a minimum of 30 min before injection of vehicle/drug. LTD was induced using an electrical LFS protocol consisting of 900 pulses run at 1 Hz, with the stimulation intensity increased to 95% maximum amplitude. A relatively weak LFS protocol (LFS-300), used to study the facilitation of LTD, consisted of 300 pulses at 1 Hz, with an intensity that evoked 95% maximum amplitude. fEPSPs were recorded for at least 1 h after LFS before a final end-run i/o curve was measured. Background EEG in the hippocampus was monitored (LabChart, AD instruments) throughout the experiment. None of the treatments had any discernable effect on baseline synaptic excitability or general EEG parameters. Upon completion of the recording session rats were perfused with 4% paraformaldehyde (Sigma) and the brain was stored at 4 °C.

### Agents

CPP ((*R*,*S*)-3-(2-carboxypiperazin-4-yl)propyl-1-phosphonic acid) (Abcam), MTEP (3-((2-methyl-1,3-thiazol-4-yl)ethynyl)pyridine hydrochloride) (Abcam), D-AP5 (D-(−)-2-amino-5-phosphonopentanoic acid) and L-AP5 (L-(+)-2-Amino-5-phosphonopentanoic acid) (both Tocris), MK-801 ((5 *S*,10 *R*)-(+)-5-methyl-10,11-dihydro-5*H*-dibenzo[*a*,*d*]cyclohepten-5,10-imine maleate) (dizocilpine) and its less active (−) enantiomer (both Tocris), and NVP-AAM077, also known as PEAQX, (tetrasodium; 5-[[[(1S)-1-(4-bromophenyl)ethyl]amino]-phosphonatomethyl]quinoxaline-2,3-diolate;hydrate) (Alamone), were prepared in ultra-clean water as stock solutions. The stock solutions were further diluted into 1 ml saline for i.p. injection; 1 ml saline was used as vehicle control. Ro 25-6981 ([R-(*R,S*)]-α-(4-Hydroxyphenyl)-β-methyl-4-(phenylmethyl)-1-piperidinepropanol hydrochloride hydrate) (Sigma) and VU 0360172 (N-Cyclobutyl-6-[2-(3-fluorophenyl)ethynyl]-3-pyridinecarboxamide hydrochloride) (Tocris) were dissolved in DMSO and diluted in saline; a 1 ml 10% v/v solution of DMSO in saline was used as vehicle control. LY341495 ((2*S*)-2-Amino-2-[(1 *S*,2 *S*)-2-carboxycycloprop-1-yl]-3-(xanth-9-yl)propanoic acid) (Abcam), was dissolved in 1.2 molar equivalent sodium hydroxide (NaOH, Sigma) and diluted in saline; saline was used as vehicle control. JNJ16259685 ((3,4-dihydro-2H-pyrano[2,3]b quinolin-7-yl) (cis-4-methoxycyclohexyl) methanone) (R&D Systems), was initially dissolved in 70% ethanol and subsequently diluted by a factor of 40 in saline.

The dose of CPP was chosen based on previous publications including our finding that 10 mg/kg, i.p., completely blocked high-frequency stimulation induced LTP^22^. The pre-injection interval was based on pilot studies and the literature^81^. In the case of MTEP, we started with a dose of 3 mg/kg, which we found completely blocked Aβ-facilitated LTD^19^. MTEP is known to achieve >90% receptor occupancy after i.p. injection with either 3 or 6 mg/kg in 10% Tween 80 in awake rats^23^. Ro 25-6981, at a dose of 6 mg/kg prevents Aβ-mediated LTP inhibition^47^. We chose doses for i.c.v. injection of Ro 25-6981 based on pilot studies and prior research^82^. The MK-801 dose of 60 nmol, i.c.v., for local injection is based on previous research^83^. (−)-MK-801 is approximately one-seventh as potent as its active enantiomer MK-801 at NMDARs^84^. The initial dose of D-AP5 was chosen on the basis of previous reports including our finding that LTP is blocked by 100 nmol after i.c.v. injection^36^. D-AP5′s stereoisomer L-AP5 is known to be at least 30-fold less potent as an NMDAR antagonist than D-AP5^37^. A dose of 3 mg/kg, i.p., LY341495 has been reported to impair recognition memory in rats^85^. This relatively low dose is used to strongly block group II mGluRs but may also at least partly block group III mGluRs^86–88^. Based on previous research^89^ and pilot studies we chose a dose of 15 mg/kg s.c. for VU 0360172. JNJ16259685 has been reported to achieve >90% receptor occupancy after s.c. injection with 0.5 mg/kg^90^. The dose of NVP was chosen based on its known ability to inhibit LTP in the range of 0.25–1 nmol after i.c.v. injection *in vivo*^47^. NVP is a competitive antagonist with an ~11-fold preference for GluN1/2 A over GluN1/2B receptors, which will likely only maintain its selectivity to block only GluN2A diheteromeric or GluN2A/B triheteromeric NMDAR-mediated synaptic responses when used at relatively low, non-saturating concentrations^77^.

### Data analysis

Values are expressed as the mean ± s.e.m. % of the baseline fEPSP amplitude measured 30 or 60 min before an injection. The magnitude of LTD was measured over the last 10 min at 1 h after LFS. Similar results were obtained when fEPSP slope was analysed. No data were excluded, and control experiments were interleaved randomly throughout. For two groups with two time points, two-way ANOVA with repeated measures with Sidak’s multiple comparison test (two-way ANOVA RM-Sidak) was used. To compare between groups or time points of three or more, one-way ANOVA with Sidak’s multiple comparisons (one-way ANOVA-Sidak) was used. Two-tailed paired Student’s *t*-tests (paired *t*) were used to compare pre- and post-LFS data within one group. A value of *P* < 0.05 was considered statistically significant.

## Electronic supplementary material

Supplementary Information
