# Supplementary material for: Physiological activation of mGlu5 receptors supports the ion channel function of NMDA receptors in hippocampal LTD induction in vivo
Source: Sci Rep. 2018 Mar 13;8:4391. doi: 10.1038/s41598-018-22768-x (PMC5849730; doi:10.1038/s41598-018-22768-x)
Supplement: Supplementary file 1 — Supplementary Information [file 41598_2018_22768_MOESM1_ESM.pdf]

SCIENTIFIC REPORTS **DOI:10.1038/s41598-018-22768-x**

Supplementary Information

Physiological activation of mGlu5 receptors supports the ion channel function of NMDA receptors in hippocampal LTD induction *in vivo*

Kenneth J. O'Riordan, Neng-Wei Hu and Michael J. Rowan

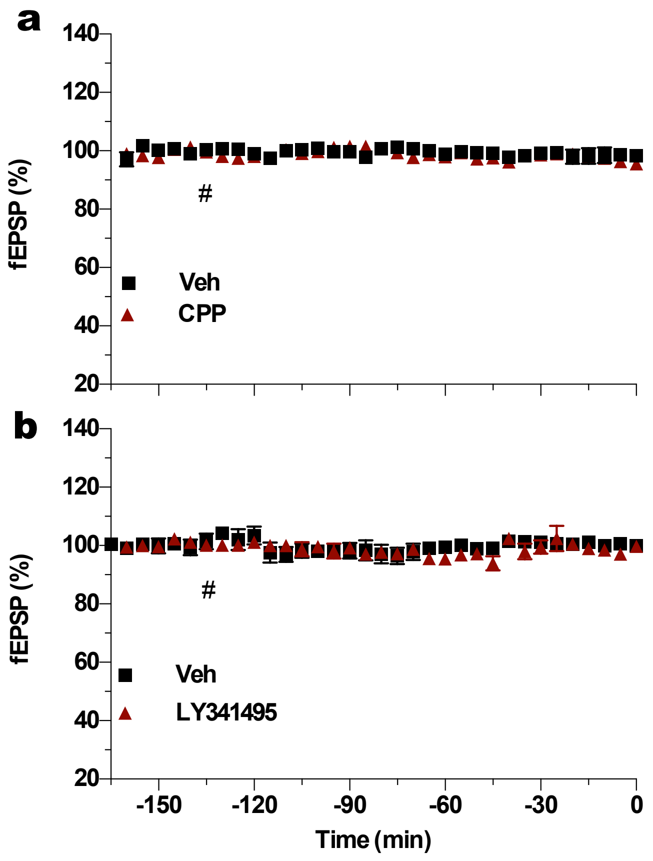

Supplementary Figure S1. Lack of effect of either the NMDA receptor antagonist CPP or the group II mGlu receptor antagonist LY341495 on baseline synaptic transmission prior to application of LFS. (**a**) Systemic administration of CPP (10 mg/kg, i.p., 2.25 h pre-LFS, hash) did not affect the amplitude of the fEPSP during the pre-LFS period (CPP: 95.9 ± 1.5%, mean ± SEM %, n = 10, $P > 0.05$ compared with vehicle, Veh: 98.4 ± 1.9%, n = 10, unpaired t-test). (**b**) Similarly, systemic administration of LY341495 (3 mg/kg, i.p., 2.25 h pre-LFS, hash) did not alter baseline responses (LY341495: 98.4 ± 1.7%, n = 8, $P > 0.05$ compared with Veh: 100.4 ± 0.9%, n = 10, unpaired t-test).

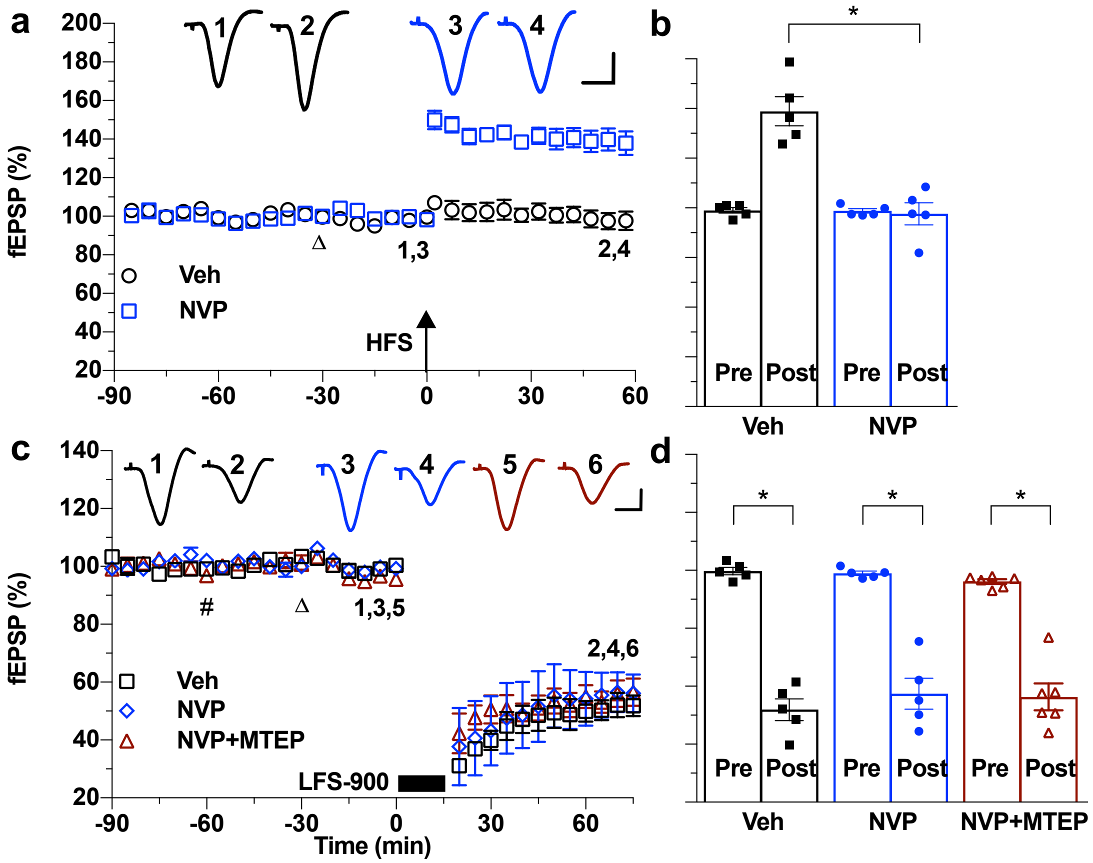

Supplementary Figure S2. The GluN2A-selective NMDA receptor antagonist NVP preferentially inhibits LTP rather than LTD. (**a**) I.c.v. injection (triangle) of 0.5 nmol (n=4) or 1 nmol (n=1) of NVP-AAM077, 30 min before HFS completely blocked HFS induced LTP (NVP: 97.7± 4.4%, n = 5; *P* > 0.05 compared with baseline and *P* < 0.05 compared with Veh: 138.9 ± 5.9%, n = 5; two-way ANOVA RM-Sidak). (**b**) Summary of the mean EPSP amplitude data in (a). (**c**) Neither NVP alone (1 nmol, n=5, triangle) nor the combination of NVP (0.5 nmol, n = 3; 1 nmol, n = 3, triangle) with MTEP (3 mg/kg, i.p., 1 h pre-LFS, hash) affected LTD induced by LFS-900. (Veh: 51.9± 3.8%, n = 5; NVP: 57.5± 5.4%; NVP+MTEP: 56.3 ± 6.4%; *P* > 0.05 compared between groups and *P* < 0.05 compared with baseline; one-way ANOVA RM-Sidak and paired *t*). (**d**) Summary of the mean EPSP data in (c). **P* < 0.05. Calibration bars: vertical, 1mV; horizontal, 10ms.
